# *Cymbopogon winterianus* (Java Citronella Plant): A Multi-Faceted Approach for Food Preservation, Insecticidal Effects, and Bread Application

**DOI:** 10.3390/foods13050803

**Published:** 2024-03-05

**Authors:** Marwa Rammal, Adnan Badran, Chaden Haidar, Abbas Sabbah, Mikhael Bechelany, Maya Awada, Khodor Haidar Hassan, Mohammad El-Dakdouki, Mohamad T. Raad

**Affiliations:** 1 Department of Food and Technology Studies, Faculty of Agronomy, Lebanese University, Beirut P.O. Box 146404, Lebanon; marwa.rammal.1@ul.edu.lb (M.R.); chaden.haidar@iul.edu.lb (C.H.); abbas.sabbah@gmail.com (A.S.); maya.awada19@gmail.com (M.A.); khodorhh@gmail.com (K.H.H.); 2 Department of Nutrition, University of Petra Amman Jordan, Amman P.O. Box 961343, Jordan; abadran12@gmail.com; 3 Institut Européen des Membranes (IEM), UMR-5635, University of Montpellier, École Nationale Supérieure de Chimie de Montpellier (ENSCM), Centre National de la Recherche Scientifique (CNRS), Place Eugene Bataillon, 34095 Montpellier, France; 4 Functional Materials Group, Gulf University for Science and Technology (GUST), Mubarak Al-Abdullah 32093, Kuwait; 5 Department of Chemistry, Faculty of Science, Beirut Arab University, P.O. Box 11-5020, Riad El Solh, Beirut 11072809, Lebanon; 6 Department of Chemistry, Lebanese International University-Beirut (LIU), Salim Salam Street, Mazraa, Beirut 146404, Lebanon; mohamad.raad01@liu.edu.lb

**Keywords:** Java Citronella, phytochemical screening, antioxidant activity, food preservative, insecticide

## Abstract

Certain plants like *Rosemarinus officinalis*, *Lavandula angustifolia* and *Origanum vulgare* have been used in the food industry for centuries. *Cymbopogon winterianus* (Java Citronella plant) is one of the most significant plants. The objective of this study is to screen for secondary metabolites by phytochemical screening, evaluate the antioxidant contents of extracts and investigate the use of the Java Citronella plant in food preservation and as an insecticide. Java Citronella powder was added to bread and evaluated for its moisture content, and a visual and sensory analysis was performed. *Sitophilus granarius* (L.) weevils were exposed to Java Citronella essential oil (JCEO). The phytochemical screening revealed that the extracts were abundant in secondary metabolites. The JCEO had a yield of 0.75%. The aqueous extract had a higher total phenolic content of 49.043 ± 0.217 mg GAE/g than the ethanolic extract, which was 24.478 ± 1.956 mg GAE/g. The aqueous extract had a total flavonoids content 27,725.25 ± 54.96 µg RE/g higher than the ethanolic extract, with 24,263 ± 74 µg RE/g. The ethanolic extract had stronger antioxidant activity, with anIC_50_ = 196.116 μg/mL higher than the aqueous extract at 420 μg/mL. The 2% Java Citronella powder in the bread was preferred by consumers, and had a shelf life of 6 days. JCEO killed all the weevils with a high dose of 10% after 48 h. The Java Citronella showed insecticidal and food preservative activity. The results should help in future research to enhance the applications of Java Citronella in various domains, from food technology to insecticides.

## 1. Introduction

Around the world, all food industries must respond to the demands of consumers and the global market by adding new ingredients to their product in order to innovate and create new products that maintain several essential aspects such as quality, safety, nutrition, shelf life and sensory characteristics [1]. Customers most often ask that they eat a healthy and natural product without the addition of synthetic preservatives that might be toxic to health or carcinogenic [2,3]. Various plants extracts have been used in food over the years as important food preservatives, owing to their antimicrobial and antioxidant attributes [3].

In this context, the Java Citronella plant (Family: Poaceae), whose scientific name is *Cymbopogon winterianus* and is also known as Citronella grass, is a common plant from Taiwan, Guatemala, Malaysia, Brazil, Ceylon, India, etc. It is spread all over the world and grows in tropical regions and hot weather. Java Citronella leaves are long, linear, leathery and light green. Citronella leaves are used as a flavoring agent and a preservative in the food, soap and fragrance industries [4]. One of the most important uses of Java Citronella plant is the extraction of its essential oil (EO) [5]. The EO of Java Citronella is sweet, light and has a strong smell that is similar to lemon. Hydro-distillation or steam-distillation methods can be employed for the extraction of Java Citronella Essential Oil (JCEO) [4]. JCEO has a repellent effect against insects due to its citronellal and citronellol compounds [6].

Bread holds a significant place in Lebanon’s daily dietary habits. However, normal bread may lack certain nutritional components, including antioxidants. Incorporating functional ingredients that are rich in antioxidants is a new method of enriching the bread with antioxidants substances [7]. Protecting bread from insects and deterioration has become one of the most important interests of food industries and the global market [8]. However, the shelf life of normal bread is very short when it is kept at room temperature for around three days [9]. Previous studies have concentrated on bread fortification with various types of plants, such as *Rosmarinus officinalis* and *Lavandula angustifolia*, and have shown improvements in moisture, nutritional components and antioxidants in comparison to regular bread [10]. Moreover, the addition of *Origanum vulgare* to bread formulations at 2% can have various effects on the final product, affecting its textural, nutritional, antioxidant, and sensory characteristics [9]. Gathering information about using these plants in bread is very important for our study because they have shown positive results in bread. These discoveries open the door for achieving the main goal of this study.

This study aims to conduct phytochemical screening to identify secondary metabolites in Java Citronella plant extracts. The study also aims to evaluate the composition and antioxidant activity of the plant in Lebanon before adding it to bread. Additionally, the investigation involves examining the impact of Java Citronella powder at different concentrations, serving as a natural food preservative, on the sensory and physical characteristics of bread. Furthermore, the study explores the insecticidal effects of JCEO on *Sitophilus granarius* (L.) weevils, over a 60 h period with varying concentrations. In this study, the Java Citronella plant was used as a food preservative in bread and as an insecticide. The powder of the Java Citronella plant was incorporated into bread instead of JCEO because it is more economic, more safe for consumption and does not have restricted use. However, JCEO was used as an insecticide because insects threaten food safety in food industries. However, until now, no study has been reported on the incorporation of Java Citronella powder on the physicochemical, visual, moisture content and sensory properties of bread.

## 2. Materials and Methods

### 2.1. Materials

Various materials and chemicals were used in this study, including the Java Citronella plant collected from the south of Lebanon (Blat–Marjaayoun) between March and June 2023. The following coordinates were detected by the Gps app: 33°23′10″ N 35°36′02″ E. Distilled water, 95% ethanol (VWR, Radnor, PA, USA), 99.90% methanol (Honeywell, Charlotte, NC, USA), Gallic acid (AnalaR), Folin reagent 10% (UNI-CHEM, Mumbai, India), sodium carbonate Na_2_CO_3_ 7% (GPR), a 2% solution of aluminum chloride in methanol (AnalaR), Gas Chromatography–Mass Spectrometry (GC-MS) employing an Agilent (Santa Clara, CA, USA) 6890N Network gas chromatograph equipped with an Agilent 19091S-433HP-5MS column, DPPH (GPR), Fehling A and B (GPR), 10% HCl (VWR), Ninhydrin 0.25% (GPR), 1% HCl (VWR), ferric chloride FeCl_3_ 1% and 5% (VWR), acetone (VWR), chloroform (VWR), concentrated sulfuric acid (VWR), 50% potassium hydroxide, KOH (UNI-CHEM), concentrated HCl (VWR), copper acetate (VWR), 10% NaOH (UNI-CHEM), concentrated H_2_SO_4_ (VWR), glacial acetic acid (VWR), K_3_(Fe(CN)_6_) 1% (AnalaR), a grinder (Compact 2100), clean plastic containers, nylon bags, a rotary evaporator Rotovap (Keyland Court Bohemia, NY, USA) (Evaporateur extracteur rotatif VV 200 Heidolph SN:129515157), an ABBE-type refractometer (serial number A8493302), Vortex (Barneveld, WI, USA) (XH-B), and a Hitachi (Tokyo, Japan) U-2900 UV-Vis spectrophotometer were used.

#### 2.1.1. Powder Preparation

The fresh leaves of Java Citronella were cleaned well and washed with distilled water, and then dried in the shade at room temperature for 1 month. After becoming completely dry, the peels were crushed and turned into powder by using a grinder. The powders were stored in clean plastic containers covered with nylon and protected from moisture in desiccators until used [10].

#### 2.1.2. Extract Preparation

The maceration technique was used to prepare the ethanolic extract and the aqueous extract separately, where 30 g of Java Citronella plant was mixed with 250 mL of pure ethanol (95%), and another 30 g of the plant was mixed with 250 mL of water distilled within a sealed container and subjected to a 24 h extraction period in darkness. The resulting extracts were then filtered using filter paper and a Buchner funnel. After the filtration technique, the ethanolic and aqueous extracts of Java Citronella were placed separately in the rotary evaporator (Rotovap) at 40 °C. In this, the solvent was evaporated while the compound remained; this was then placed in a freezer at −80 °C before being placed in a lyophilisation machine. The powders obtained for each extract were stored in clean plastic containers covered with nylon and protected from moisture in desiccators until used [10].

#### 2.1.3. Essential Oil Extraction

The Java Citronella essential oil (JCEO) extraction involved hydro-distillation with a Clevenger-type apparatus over a 4 h duration. The cut fresh leaves of the plant were placed in a flask with 4 L of distilled water, heated, and the vapors were condensed in a cooler, causing the oils to separate from the water due to density differences. The EO was kept in an amber glass bottle at a temperature of 4 °C until it was needed [10]. The EO yield was determined by calculating the ratio of the volume of essential oil to the mass of the plant material.

$$Yield\left(\%\right)=\frac{V_{HE}}{M_{VS}}\times 100$$

where $V_{HE}$ is the volume of essential oil (mL) and $M_{VS}$ is the mass of plant material (g).

### 2.2. Relative Density of the Oil

The relative density of JCEO was assessed in accordance with ISO 279:1998 [11] by first measuring the mass of 1 mL of the essential oil at 20 °C. This way, the density (ρ20), which is the mass of a unit volume of the essential oil at 20 °C, was obtained using the following equation [10]:

$$\mathrm{Relative}\;\mathrm{density}\;\mathrm{of}\;\mathrm{the}\;\mathrm{essential}\;\mathrm{oil}=\frac{1}{\mathrm{relative}\;\mathrm{density}\;\mathrm{of}\;\mathrm{water}\;\mathrm{at}\;20\;{}^{\circ}C}\times \rho 20=\;1.00180\times \rho 20$$

### 2.3. Refractive Index

The refractive index of the JCEO was determined by using the refractometer, which is a handheld, analog instrument with a unit Brix at 20 °C.

### 2.4. Gas Chromatography (GC) Analysis

Gas Chromatography–Mass Spectrometry (GC-MS) was conducted to identify the compounds in the extracted JCEO. This analysis used an Agilent 6890N Network Gas Chromatograph equipped with an Agilent 19091S-433HP-5MS column, with dimensions of 30 m × 0.25 mm × 0.25 µm. First, 1 µL of the essential oil was injected into the GC inlet, and then a column flow rate of 1.3 mL/min was maintained. Initially, the column temperature was established at 325 °C, and the injection temperature was set to 280 °C. The oven temperature was programmed to increase from 65 °C to 450 °C at a rate of 3 °C per min. Subsequently, the detector scanning was conducted for a duration of 45 min. The identification of the reported individual components was achieved through a comparison of the mass spectra and by referring to the GC-MS library.

### 2.5. Qualitative Study—Phytochemical Screening

The chemical composition of the Java Citronella ethanol extract and aqueous extract was determined by the qualitative tests shown in Table 1. Thus, the biological activity of the Citronella Java ethanol extract and aqueous extract could be estimated thanks to these reactions, which highlight the presence of the primary or secondary metabolites responsible for certain biological effects.

### 2.6. Quantitative Study

#### 2.6.1. Total Phenolic Content (TPC)

##### Preparation of the Standard Gallic Acid Curve

A standard curve was established utilizing Gallic acid as a reference, and the Farhan method was used with slight adjustments [20]. Serial dilution from a stock solution with a concentration of 400 μg/mL was performed to prepare different concentrations of Gallic acid solution (1.5625; 3.125; 6.25; 12.5; 25; 50 μg/mL). The Gallic acid powder was dissolved in distilled water. Then, 100 μL of each Gallic acid concentration was combined with 1000 μL of 10% Folin reagent and left to incubate for 5 min. After that, 1 mL of 7% sodium carbonate (${Na}_{2}{CO}_{3}$) was added, and the mixture was mixed by using vortex before undergoing a 30 min incubation in the dark at room temperature. The blank consisted of 100 μL of distilled water, 1000 μL of 10% Folin Ciocalteau, and 1 mL of 7% ${Na}_{2}{CO}_{3}$. The absorbance of the solutions was measured at 765 nm using the Hitachi U-2900 UV-V spectrophotometer.

##### Preparation of Ethanolic/Aqueous Extracts

The Java Citronella ethanolic and aqueous extracts, each comprising a volume of 100 μL, were taken separately and mixed with 1000 μL of the Folin reagent; the mixture was then incubated in darkness for 5 min. Then, 1000 μL of sodium carbonate ${Na}_{2}{CO}_{3}$ 7% was added to each solution, mixed by using a vortex and left to incubate in the dark at room temperature for 30 min. This procedure was repeated three times. The blank was composed of 100 μL of solvent (ethanol 95%/distilled water), 1000 μL of Folin Ciocalteau and 1 mL of ${Na}_{2}{CO}_{3}$ 7%. The absorbance of all samples was measured at 30 min and recorded using the Hitachi U-2900 UV-Vis spectrophotometer at 765 nm. The total phenolic content was calculated using the following formula:

$$\mathrm{TPC}\;(\mathrm{mg}\;\mathrm{GAE}/g)=\frac{\mathrm{GAE}\;\times \;V\;\times \;D}{m}$$

where GAE (mg/mL) is calculated by projecting the Optical Density (O.D.) values on the gallic acid standard curve (line equation), V is the sample volume in mL, D is the dilution factor, and m is the weight in g of plant extract.

##### 2.6.2. Total Flavonoid Content (TFC)

###### Preparation of the Standard Rutin Curve

The aluminum chloride method, as outlined by Quetier-deleu [21], was employed with slight adjustments. Various concentrations of the extracts (1 mL) were mixed with 1 mL of 2% methanolic aluminum chloride solution. Following a 30 min incubation period at room temperature in the dark, the absorbance of the samples was measured at 415 nm using a Hitachi U-2900 UV-Vis spectrophotometer. The results were expressed in mg per g of rutin equivalent (RE), with methanol serving as the blank.

###### Preparation of Ethanolic/Aqueous Extracts

In this step, 1000 μL of the Java Citronella ethanolic/aqueous extract was taken and mixed with 1000 μL of the aluminium chloride by using vortex and left to incubate in the dark at room temperature for 30 min. This procedure was repeated three times. The blank was composed of 1 mL of solvent (ethanol 95%/distilled water) and 1 mL of the aluminum chloride. The absorbance of each sample was measured at 415 nm using the Hitachi U-2900 UV-Vis spectrophotometer. The total flavonoid content was determined using the following formula:

$$\mathrm{TFC}\;(µg\;\mathrm{RE}/g)=\frac{RE\times V\times D}{m}$$

where RE (µg/mL) is calculated by projecting the Optical Density (O.D.) values on the rutin standard curve (line equation), V is the sample volume in mL, D is the dilution factor, and m is the weight in g of plant extract.

#### 2.7. Evaluation of Anti-Oxidant Activities

##### Preparation of Ethanolic and Aqueous Extracts

The method of Rammal [22] was used to test the scavenging ability of DPPH, with slight modifications. First, 1 mL of different concentrations (0.1, 0.2, 0.4, 0.6, 0.8 and 1 mL) of diluted ethanolic and aqueous extracts of the Java Citronella was added to 1 mL of DPPH (2.5 mg in 50 mL methanol); at the same time, a control consisting of 1 mL of DPPH with 1 mL of pure ethanol (95%) for the ethanolic extract and 1 mL of distilled water for the aqueous extract was prepared. After mixing them by using vortex, they were left to incubate in the dark at room temperature for 30 min. The experiments were conducted in triplicate. The blank was composed of 2 mL of methanol and 2 mL of ethanol 95% for the ethanolic extract, and for the aqueous extract, the blank was composed of methanol and 2 mL of distilled water. The absorbance of all samples was then measured at 517 nm using the Hitachi U-2900 UV-Vis spectrophotometer. The percentage of DPPH radical scavenging by the various samples was determined using the following formula:

$$\mathrm{Percentage}\;\mathrm{of}\;\mathrm{inhibition}=\frac{\mathrm{absorbance}\;\mathrm{of}\;\mathrm{control}-\;\mathrm{absorbance}\;\mathrm{of}\;\mathrm{the}\;\mathrm{extract}}{\mathrm{absorbance}\;\mathrm{of}\;\mathrm{control}}\times 100$$

#### 2.8. Food Preservative

##### 2.8.1. Raw Materials for Bread Preparation

Whole wheat flour category 65 (Naddour Wheat Flour, Beirut, Lebanon), salt, dry yeast, and water [23].

##### 2.8.2. Bread Making Technology

To determine the influence of Java Citronella powder on the quality of flat bread, powdered leaves of Java Citronella were incorporated into the mixture at concentrations of 0.5%, 1%, 1.5%, 2%, 2.5% and 3% of the total whole wheat flour content. A control bread (©) was utilized for comparison, with no powdered leaves added to the mixture. All the ingredients were mixed for approximately 6 min at a minimum speed using a dough mixer. The dough samples were then left to ferment at room temperature for 2 h. The bread samples were then baked at a temperature of 115 ± 5 °C with 60% relative humidity for 3 min each on a heated oven. They were then cooled to room temperature, specifically 22 ± 2 °C, for a duration of 2 h. After that, they were packaged using a nylon bag at 20 ± 2 °C with 48% relative humidity [23].

##### 2.8.3. Visual Analysis of Bread

The bread samples were incubated at room temperature in a nylon bag (to preserve moisture in bread). The bread samples were observed for 8 days for mold detection [9].

##### 2.8.4. Bread Moisture Content

The moisture content of the bread was assessed by measuring the mass loss of the bread samples, which were oven dried at 105 ± 5 °C until a consistent mass was achieved on day 1, day 4, and day 8. The breads samples were packaged in a nylon bag after drying [23]. The process was replicated three times. The moisture content was determined using the following formula:

$$MC=\frac{mi-mf}{mi}\times 100$$

*mi* = initial mass of bread before drying

*mf* = final mass of bread after drying

##### 2.8.5. Sensory Evaluation of the Bread

At the pilot scale, a hedonic sensory analysis was conducted at the Lebanese University—Faculty of Agricultural Engineering and Veterinary Medicine with 60 panelists (appearance, texture, odor, color, flavor, mouth feel, after taste, overall acceptability), where participants aged between 18 and 60 provided their feedback using a 9-point hedonic scale, where 1 denoted extreme dislike and 9 indicated extreme liking; the scale was labeled ‘Dislike Extremely’ on the left, ‘Like Extremely’ on the right, and ‘Neither Like nor Dislike’ in the center. All samples were coded using three-digit, randomly generated numbers and served in the shape of small triangles; the samples were placed in similar containers labeled with randomly assigned 3-digit codes. Each panelist was provided with a glass of water to mitigate carry-over effects.

At the laboratory scale, different from the control test (DFC), tests were performed after one day of baking with 7 laboratory panelists at the “Agroalimentaire Laboratory”, which is part of the Faculty of Agricultural Engineering and Veterinary Medicine at the Lebanese University. A scale of 0 (no difference) to 5 (very large difference) was used to evaluate the difference between the control and the most acceptable sample by the consumer. The panelists were provided with triangular-shaped samples that were placed in similar containers labeled with randomly assigned 3-digit codes. Each panelist was provided with a glass of water to mitigate carry-over effects. Informed consent was obtained from all volunteers involved in the sensory study.

#### 2.9. Insecticide

##### 2.9.1. Equipment

The equipment used in this experiment was as follows: 6 test tubes, 7 petri dishes with a diameter of 90 mm, 2 micropipettes of ranges 5–40 µL and 40–200 µL, a clean tray, a spatula and a dropper.

##### 2.9.2. Materials

The materials used were as follow: 70 grains of *Sitophilus granarius* (L.) weevils (10 weevils in every petri dish), JCEO, ethanol and 210 Freekeh beans (30 seeds in each petri dish).

##### 2.9.3. Procedure

###### Preparation of Dishes

The *Sitophilus granarius* (L.) weevils were incubated and left to reproduce and replicate in an environment convenient for use in this experiment. All the Petri dishes with a diameter of 90 mm were purchased, and 10 *Sitophilus granarius* (L.) weevils were placed in each petri dish; these were then closed firmly. Each dish was labelled with specific notations that abbreviate the concentration of solutions to be prepared next. In this study, the effect of JCEO was studied at percentages of 2, 3, 5, 7, and 10% JCEO. NA: control (only contains weevils), ET: ethanol.

##### Preparation of Solution

The solutions with varying concentrations (2%, 3%, 5%, 7%, and 10%) of EO were prepared in 5 test tubes that were cleaned thoroughly and left to dry for 6 min at room temperature. The sets of solutions contained different types and portions of EO mixed with ethanol to make each solution up to 1 mL. Every tube was effectively sealed with a nylon covering to inhibit EO evaporation. Then, each solution was positioned within labeled Petri dishes that contained 10 *Sitophilus granarius* (L.) weevils and left at room temperature 20 °C for a certain period of time: 30 min, 6 h, 24 h, 48 h, or 60 h. Pictures were captured at regular intervals to observe the results.

The set containing Java Citronella essential oil:



**Tube 1**

**Tube 2**

**Tube 3**

**Tube 4**

**Tube 5**

**Tube 6**

**Tube 7**

ControlET2% JCEO3% JCEO5% JCEO7% JCEO10% JCEOEO/ET ratio (*v/v*)-0/1000 μL20/980 μL30/970 μL50/950 μL70/930 μL100/900 μL

#### 2.10. Statistical Analysis

A statistical analysis was performed using Excel version 2013 for the bread moisture content, bread sensory analysis, DFC test and for the determination of JCEO TL50. To study the variation in the overall acceptability of the bread samples and to determine the LD50 of JCEO, the statistical One-way ANOVA test was employed, using STATISTICA 64 to compare the means of the panellist’s response.

## 3. Results and Discussion

### 3.1. JCEO Extraction

The characteristics of JCEO in this study are in accordance with the ISO 3848:1976 standards (Table 2) [24]. The color of JCEO produced in this study was a pale brownish-yellow- color, where the yellow color is due to the carotene substance. In this study, as the density and refractive index are high, the JCEO has a good quality and high yield [24].

The yield of JCEO obtained in the current study is 0.75% higher than the JCEO yield obtained in Nepal, which was 0.5%. The variation in the yield of essential oils from different origins could be linked to harvesting periods, climates, and growth environments [6].

### 3.2. Isolation and Chemical Composition of the Essential Oil

The Gas Chromatography/Mass Spectrometry (GC/MS) revealed the detection of 50 different compounds. The gas chromatogram illustrating the JCEO is presented in Table 3. Table 3 provides details on the identified compounds in the sample oil, including their retention time and area percentage. The most important chemical components of JCEO are as follows: geraniol, citronellal and citronellol. Citronellal and citronellol are responsible for repelling insects such as mosquitoes and for the lemony smell. Geraniol provides the antimicrobial, antioxidant, and anti-inflammatory activity. In this study, the JCEO had a citronellal (32.611%) content higher than that obtained in the other study performed in Nepal, at 11.85%, a geraniol (25.946%) content lower than that in the other study (28.87%), and a citronellol (17.540%) content higher than that in the other study (10.88%) [6]. The components listed in Table 3 represent the principal constituents identified in the JCEO.

### 3.3. Qualitative Test—Phytochemical Screening

The aqueous extracts and ethanolic extracts of Java Citronella obtained by the maceration technique are rich in the primary and secondary metabolites represented in Table 4.

The presence of various phytochemicals in Java Citronella extracts, as indicated in Table 4, aligns with known studies and the literature on this plant species. Phytochemical screenings aim to identify the various secondary metabolites present in plants, which can vary based on the solvent used for extraction.

The phytochemical screening, as shown in Table 4, showed that the aqueous extracts contained the following: reducing sugars, anthraquinones, tannins, resins, terpenoids, flavonoids, quinones, sterols and steroids, flavanones, phenols, fixed oils and fatty acids.

Reducing sugars are often found in many plants and used for energy. Anthraquinones are recognized for their healing properties and encountered in certain medicinal plants. Tannins are known for their antioxidant properties, and are commonly found in various plant extracts. Resins comprise intricate combinations of terpenoids and phenolic compounds. Terpenoids are common secondary metabolites with diverse biological activities. Flavonoids are known for their antioxidant and anti-inflammatory properties. Quinones have diverse biological activities, including antimicrobial properties. Sterols and steroids are important constituents with various physiological effects. Flavanones, phenols, fixed oils and fatty acids are found in plant extracts [25].

The ethanolic extracts phytochemical screening affirmed the presence of the following: reducing sugars, anthraquinones, proteins and amino acids, phlabotannins, terpenoids, flavonoids, quinones, sterols and steroids, diterpenes, flavanones and cardiac glycosides. These align with the aqueous extract results, indicating some consistency. Proteins and amino acids are essential components found in most plant tissues. Phlobatannins possess astringent characteristics and can be found in different medicinal plants. Diterpenes and cardiac glycosides are known for their diverse biological activities. The presence of these phytochemicals in different solvents corroborates the understanding that the choice of solvent influences the types of compounds extracted from plants. Ethanol is known for its ability to dissolve a wide range of phytochemicals, while water might selectively extract certain compounds [25]. The consistency in the presence of compounds like reducing sugars, anthraquinones, terpenoids, flavonoids, and sterols across both extracts aligns with established knowledge about the Java Citronella plant. However, specific variations might occur due to environmental factors, plant maturity, extraction techniques, and geographical locations, impacting the phytochemical profile of plant extracts [10].

### 3.4. Quantitative Test

#### 3.4.1. Total Phenolic Compounds (TPC)

**Figure 1:**
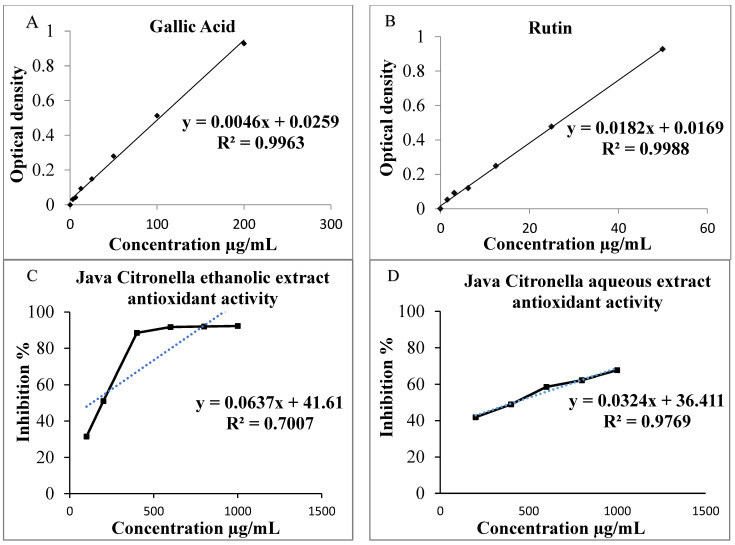
(**A**) Gallic acid calibration curve to assess TPC; (**B**) Rutin calibration curve to assess TFC; (**C**) Antioxidant activity of Java Citronella ethanolic extract evaluated through the DPPH radical scavenging test; (**D**) The antioxidant activity of Java Citronella aqueous extract measured using the DPPH radical scavenging test.

The Gallic acid equivalence (GAE) of each extract is determined according to the curve in mg/mL from the equation y = 0.0046x + 0.0259; $R^{2}$ = 0.9963 (Figure 1A). For the Java Citronella aqueous extract, the total phenolic content is 49.043 ± 0.217 mg GAE/g of dry extract, surpassing the total phenolic content in the ethanolic extract of Java Citronella, which measures at 24.478 ± 1.956 mg GAE/g of dry extract.

#### 3.4.2. Total Flavonoids Content (TFC)

The Rutin equivalent (RE) of each extract is determined according to the curve in μg/mL from the equation y = 0.0182x + 0.0169; $R^{2}$ = 0.9988 (Figure 1B). The total flavonoid content for the Java Citronella aqueous extract is 27.725 ± 55 µg RE/g of dry extract, surpassing the total flavonoid content for the Java Citronella ethanolic extract, which measures at 24.263 ± 74 µg RE/g of dry extract.

#### 3.4.3. DPPH Antioxidant Activity

Java Citronella extracts have dose-dependent antioxidant activity, as shown in Figure 1C,D; when the concentration of Java Citronella aqueous extract and ethanolic extract increases from 200 to 1000 μg/mL and from 100 to 1000 μg/mL, respectively, the inhibition percentage rises as well, reaching its peak activity. At 1000 μg/mL, the ethanolic extract has a maximum inhibition percentage of 92.281%, which is higher than that of the aqueous extract, at 68%. In addition, the IC_50_ of each sample is determined according to the graph by linear regression. The IC_50_ is the concentration at which each sample reaches 50% of its maximum DPPH radical inhibitor activity. The IC_50_ values exhibit an inverse relationship with the antioxidant activity; thus, the greatest antioxidant efficacy is linked with the lowest IC_50_ concentration. The outcomes presented in Table 5 reveal that Java Citronella possesses antioxidant activity, with an IC_50_ of 420 μg/mL for the aqueous extract; this is higher than the IC_50_ for the ethanolic extract, which is 196.116 μg/mL. This suggests that the ethanolic extract demonstrates higher antioxidant activity than the aqueous extract. The results indicate strong radical scavenging activity for both the Java Citronella ethanolic and aqueous extracts.

### 3.5. Bread Making Technology

#### 3.5.1. Bread Visual Analysis over Time

**Figure 2:**
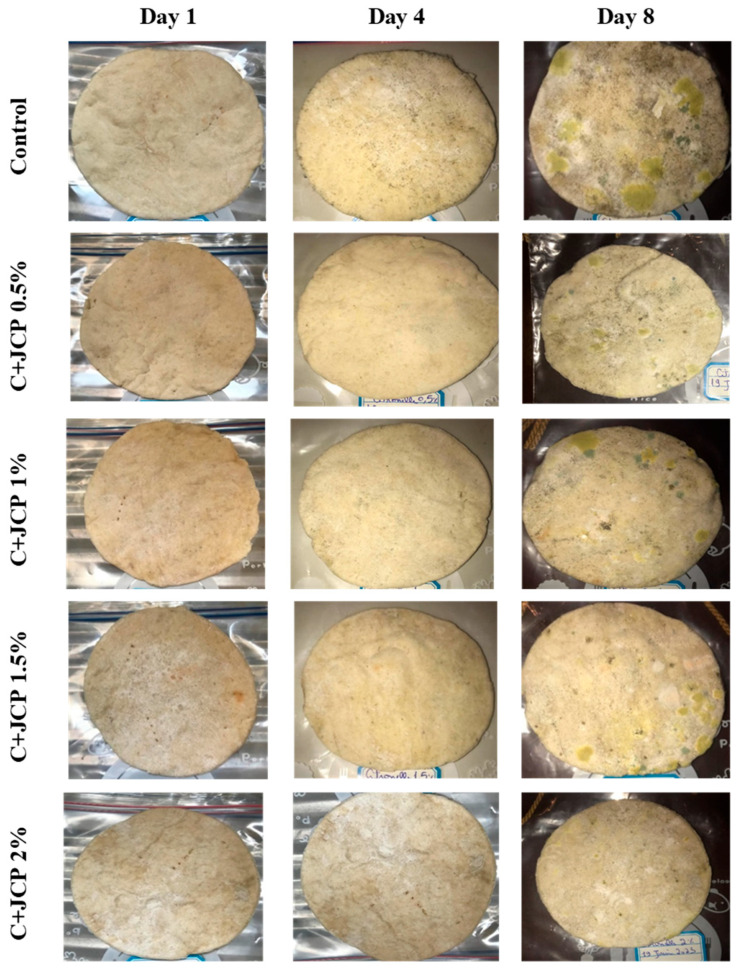
Bread containing varying concentrations of powdered Java Citronella plant.

A visual analysis of the bread, as shown in Figure 2, demonstrated that less fungal growth appeared as the concentration of Java Citronella powder in the bread increased. The bread samples with 2%, 2.5% and 3% Java Citronella powder showed less mold growth than the other bread samples. However, the control bread sample revealed the most mold growth between all bread samples.

Table 6 shows that bread prepared with 2%, 2.5% and 3% Java Citronella powder had a shelf life of 6 days. The control bread, which contained no Java Citronella powder, spoiled in 3 days. Java Citronella powder showed a positive effect on preventing microbial and fungal growth on bread, and the incorporation of 2%, 2.5% and 3% Java Citronella powder prevented the bread from being attacked by fungi for 6 days at room temperature.

These findings align with the study conducted by Dhillon, which demonstrated that incorporating 2%, 3%, and 4% oregano, known for its potential antimicrobial properties, extended the shelf life of bread by inhibiting the growth of spoilage microorganisms, resulting in a 6-day extension at room temperature [9].

#### 3.5.2. Bread Moisture Content

**Figure 3:**
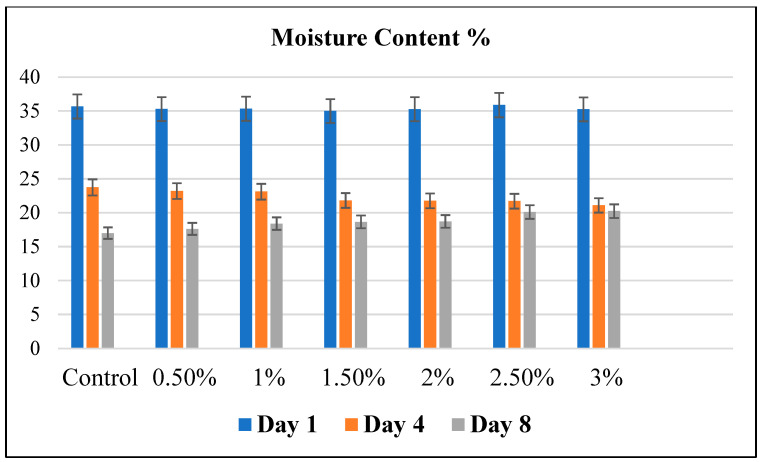
The moisture content of bread fortified with Java Citronella powder by using the oven drying method. Statistical analysis is shown using the Statistica program in the Supporting Information (Table S1 day 1, Table S2 day 4 and Table S3 day 8).

Figure 3 shows the moisture content of bread fortified with Java Citronella powder on day 1, day 4 and day 8.

The values are presented as the mean ± standard deviation (SD) of the samples.

The ideal moisture content range for a standard moist bread during a one-day storage period is typically between 35% and 45% [7]. According to Figure 3, the moisture content of all bread samples was between 35.007 ± 0.611% and 35.87 ± 0.232%. The moisture content of all bread samples diminished throughout the 8-day storage period post-baking. This may be due to the crystallization process that leads to a syneresis mechanism where moisture migrates to the surface of bread, which causes the staling of bread and moisture loss. The rate of moisture loss is impacted by the relative humidity of the storage environment. The growth of microbes in food cannot be solely explained by the moisture content [26]. The loss of moisture may transpire through evaporation both during the baking process and throughout the storage period [7].

An elevated moisture content enhances vulnerability to microbial growth and enzyme activity. On day 4, the moisture content, as shown in Figure 3, was highest in the control bread (23.74% ± 0.61). It decreased as the Java Citronella plant’s concentration increased to 21.08% ± 0.23 with the 3% Java Citronella bread. This explains the slight growth of mold in the control and low-concentration bread powdered with the Java Citronella plant.

The presence of moisture in bread is a crucial element that adds moisture and lubrication to the bread. When the moisture is too low, it decreases the firming and the bread becomes hard [7]. On day 8, the moisture content of the bread incorporated with Java Citronella powder was higher compared to the control bread; this can be explained by the increased capacity to retain moisture due to the incorporation of Java Citronella powder. Higher Java Citronella concentrations might result in increased moisture retention in bread. This outcome indicates that the incorporation of Java Citronella powder has a beneficial impact on the moisture content of the bread. These findings align with the observations from the visual analysis test.

#### 3.5.3. Bread Sensory Analysis

Overall acceptability is one of the most important sensory criteria because it shows us the bread considered most acceptable by the consumers through a hedonic scale, where the panelists indicate how much they accepted and preferred the new product.

**Figure 4:**
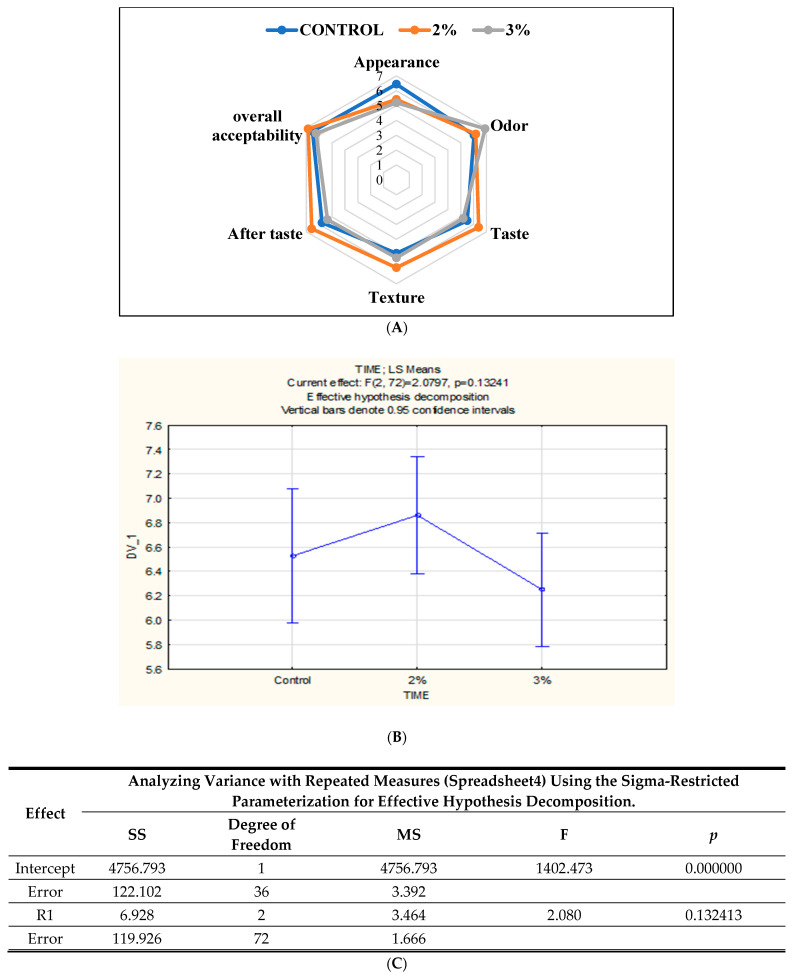
(**A**) Radar plot showing the sensory profile obtained for the three samples of bread tested; (**B**,**C**) The sensory evaluation of the bread’s overall acceptability through hedonic analysis.

According to the results in Figure 4B, the 2% Java Citronella bread sample had a higher acceptability rate of 6.88 than the control bread and 3% Java Citronella bread. There is no significance difference between the samples (*p* > 0.05).

Sensory evaluation was conducted to assess the sensory acceptance of the bread enhanced with powdered plants. As observed in Figure 4A, the bread incorporated with Java Citronella at the 2% level had a higher acceptability rate than the control and 3% Java Citronella bread. The overall acceptability score varied from 6.25 to 6.88. The results of the sensory analysis suggested that the addition of the Java Citronella plant in bread had a positive response towards consumer acceptability. The incorporation of Java Citronella had a significant effect on the texture (elasticity) of bread, where the 2% Java Citronella bread was more preferred than the other bread samples. The elasticity of bread is linked to its moisture content. However, the moisture content of the bread samples on day 1 was nearly identical. The appearance (color) of the control bread was rated the highest. It seems that consumers preferred the light color of normal bread because the addition of Java Citronella powder can slightly reduce the light color of bread. Odor was the highest with the 3% Java Citronella bread, followed by the 2% Java Citronella bread and then the control bread. The smell of Java Citronella powder is very strong, and it leaves a sweet and lemon-like odor. According to the taste analysis, the 2% Java Citronella bread had the highest accepted value. It showed a better taste and aftertaste than the control and 3% bread.

**Figure 5:**
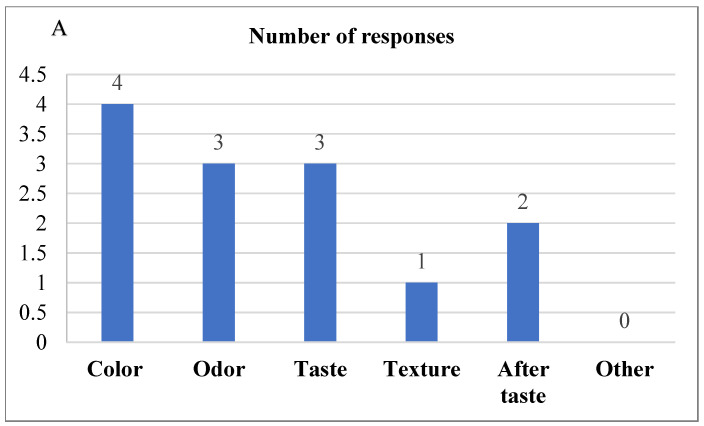

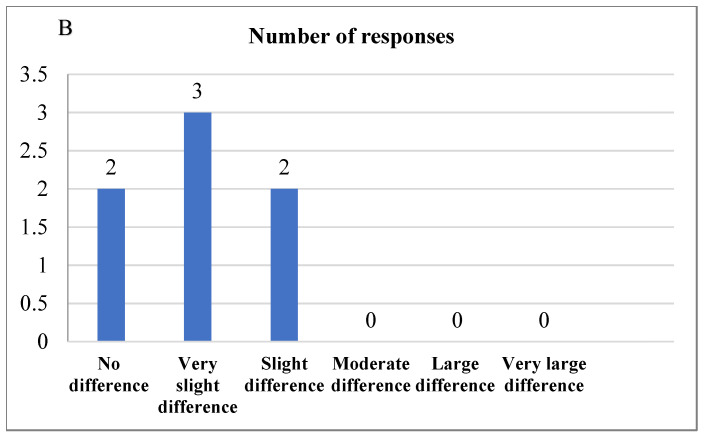
(**A**) DFC test reveals significant differences in color, odor, taste, texture, and aftertaste between control and 2% bread samples according to laboratory panelists; (**B**) DFC test indicates slight differences in control and 2% bread samples as noted by laboratory panelists.

The difference from the control test was evaluated to compare the difference between the control bread and the 2% Java Citronella bread, which was the bread most accepted by the consumer. The level was at no difference (score = 0) to slight differences (score = 2) between the samples (Figure 5). The laboratory panelists noticed slight differences between the control bread and the 2% Java Citronella bread in color, odor, taste, texture and aftertaste. The color had the highest number of responses, where panelists detected a slight difference in color.

These findings are consistent with the findings reported by Dhillon, suggesting that incorporating 2% oregano at this concentration does not result in significant changes in the baking and sensory properties of the bread. However, it potentially enhances the shelf life of the bread, as indicated by results from other studies [9].

### 3.6. Insecticide

#### 3.6.1. Effect of the Insecticide on the Weevils

To note the mortality of weevils after 60 h, images were taken at each interval to note the results obtained after the application of different % of JCEO on the weevils after 30 min, 6 h, 24 h, 48 h and 60 h, as indicated in Table 7.

#### 3.6.2. Determination of Lethal Time 50 (LT_50_)

**Figure 6:**
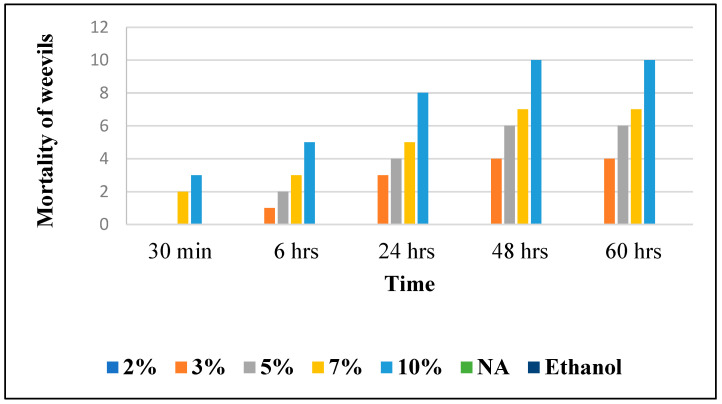
Effect of JCEO on weevil’s mortality over time.

The LT_50_ (the time after which 50% of the insects die following the application of different products) was determined based on Figure 6 in order to assess the effectiveness of the treatments applied; this is clearly stated in Table 8.

#### 3.6.3. Determination of Lethal Dose 50 (LD_50_)

**Figure 7:**
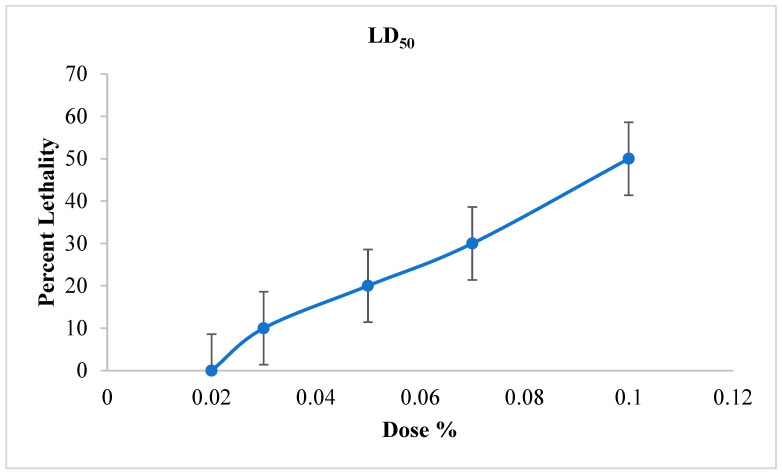
Effect of Java Citronella essential oil on weevils’ mortality over time.

The LD_50_ (the dose of product capable of causing 50% death after 6 h) was determined based on Figure 7 in order to assess the effectiveness of the treatments applied.

According to this figure, the LD_50_ % of JCEO is 10%. It should be noted that the insecticide effect should depend on two important factors: the concentration of the supplemented extract and the duration of the experiment. According to Figure 6:

At t = 30 min: 2%, 3%, and 5% of all the essential oil doses showed no lethal effect, while 7% JCEO showed a lethal dose (20%) and that of 10% showed higher lethality (30%) because of its high relative concentration.

At t = 6 h: 2% JCEO showed no lethality at this dose, 3% JCEO increased to 10% lethality, 5% increased to 20% lethality and 10% JCEO showed 50% lethality as it reached its IC_50_; less than 7% still showed low mortality (30%).

At t = 24 h: JCEO (3% to 10%) showed an increase, respectively, in the mortality percentage; 3% JCEO showed a 30% mortality, 5% JCEO showed a 40% mortality, 7% JCEO showed a 50% mortality, while 10% JCEO showed an 80% mortality at this time.

At t = 48 and 60 h: 2% JCEO showed no mortality during this time, 3% showed an increase in mortality of 40%, 5% showed an increase to 60% mortality, 7% showed a 70% mortality and 10% JCEO showed maximum lethality (100%). In addition, 10% of JCEO is a very high concentration and it took a long time (48 h) to kill all the *Sitophilus granarius* (L.) weevils, which means that JCEO may not be very effective against this type of weevil but that it is toxic in a high dose. However, the Cymbopogon Winterianus plant presents insecticide activity, which could be due to the constituents contained in JCEO, such as citronellol and citronellal [6]. The EO from clove led to the 100% mortality of grain weevils 48 h post-treatment at the specified concentrations of 17.9 and 35 μL/g, showing a higher efficiency against weevils than the Java Citronella plant [26].

These results are consistent with what has been documented by Hamed et al., which demonstrates that a 7% concentration of lavender essential oil (LEO) leads to a 50% mortality rate in weevils within a short duration of 6 h. Additionally, when combining rosemary essential oil (REO) and LEO at 5.5%, there is a reduction in LD50 (lethal dose for 50% mortality) from 9% with REO alone and 7% with LEO alone. This suggests that the synergistic effect of these essential oils is more potent at lower concentrations compared to their individual use. In conclusion, the combination of REO and LEO at a concentration of 5.5% appears to be the most efficient in achieving a mortality rate of over 50% in weevils [10].

## 4. Conclusions

The phytochemical screening of Java Citronella leaves revealed significant secondary metabolite, total phenol, and flavonoid contents, emphasizing its antioxidant-rich nature. The incorporation of Java Citronella powder at a 2% concentration demonstrated a preservative effect in bread, which was well received by consumers. Additionally, the JCEO exhibited insecticidal properties against Sitophilus granarius weevils, with a 10% solution causing a notable mortality rate within 6 h. Our study successfully achieved its objectives, presenting novel insights into the use of Cymbopogon winterianus powder in bread. Comparable to the literature findings, the incorporation of oregano, lavender, and rosemary in bread also showed preservative and insecticidal effects [9,10]. Future research should explore improving the appearance of Java Citronella-incorporated bread, testing various plant powders, and scaling up for industrial use. These results contribute to expanding the applications of Java Citronella in food technology and insecticides. Further investigations are warranted to optimize the final product on a larger industrial scale, study the antioxidant activity of supplemented bread and enhance its applications in diverse domains.

## Figures and Tables

**Table 1:** Qualitative identification of primary and secondary metabolites in JCEO.

Metabolites	Extract	Reagent	Colour	Reference
Reducing sugar	0.5 mL	1 mL of water + 5 drops of Fehlings (A + B) + boil 5 min	Brick-red precipitate	[12]
Anthraquinones	1 mL	1 mL of HCl (10%) + boil 5 min	Precipitate	[13]
Proteins and amino acids	1 mL	1 mL of Ninhydrin (0.25%) + boil 5 min	Blue colour	[14]
Phlabotannins	1 mL	1 mL of HCl (1%) + boil 5 min + cooling	Red precipitate	[15]
Tanins	1 mL	1 mL of rerric chloride FeCl_3_	Blue colour	[16]
Resins	1 mL	Acetone + small amount of water	Turbidity	[17]
Terpenoïds	1 mL	2 mL of chloroform + 3 mL of concentrated sulfuric acid	Reddish brown colour in the surface	[12]
Flavonoïds	1 mL	5 mL of potassium hydroxide KOH (50%)	Yellow colour	[18]
Quinones	1 mL	1 mL HCl concentrated	Precipitate or yellow colour	[13]
Sterols and steroids	1 mL	2 mL of chloroform + concentrated sulfuric acid	Red colour of the upper layer + greenish yellow fluorescence in the acid layer	[11,17]
Diterpenes	1 mL	Few drops of copper acetate	Green colour	[16]
Anthocyanins	1 mL	1 mL of NaOH (10%)	Blue colour	[19]
Flavanones	1 mL	${1mL\;of\;H}_{2}{SO}_{4}$ concentrated	Purple red colour	[19]
Cardiac glycosides	2 mL	1 mL of glacial acetic acid + 1 drop of 5% ferric chloride (FeCl_3_) + 1 mL of concentrated sulfuric acid	Purple ring + Brown ring + Green ring	[11,17]
Saponins	2 mL	Vigorous shaking (5 min on Vortex)	Layer of foam	[12]
Phenols	5 mL	1 mL of ${FeCl}_{3}$ (1%) + 1 mL of $K_{3}{\left(Fe(CN\right)}_{6}$) (1%)	Greenish blue colour	[16]
Fixed oils and fatty acids	Small amount of extract	On filter paper	Oil spot	[12]

**Table 2:** Physical properties of Java Citronella essential oil.

Parameters	Result of This Study	ISO 3848:1976
Color	Pale brownish-yellow- color	Pale brownish-yellow color
Density	0.914	0.880–0.922
Refractive Index	1.47	1.466–1.475

**Table 3:** GC/MS of JCEO obtained by hydrodistillation.

Peak	Compound Name	Retention Time (min)	Area Percentage %
1	Limonene	4.691	2.118665
2	Cyclohexanol, 1-(aminomethyl)-	4.841	0.047787
3	1,3,6-Octatriene, 3,7-dimethyl-, (E)-	5.791	0.142653
4	2-Pyridinemethanamine, N-methyl-	6.076	0.084911
5	Bicyclo [3.1.0] hexan-2-one, 3,3,6-trimethyl-	6.258	0.050981
6	cis-Linaloloxide	6.839	0.062695
7	Bicyclo [3.1.0] hexane, 6-methylene-	7.456	0.18315
8	Geranial	7.742	1.252344
9	Cyclohexene, 3,3,5-trimethyl-	8.971	0.144248
10	6-methyl-Hept-5-en-2-one	9.231	0.426766
11	3,3-Dimethyl-hepta-4,5-dien-2-one	9.376	0.324244
12	Eucalyptol	9.516	0.114286
13	Cyclooctane, ethenyl-	9.973	0.76096
14	Cyclooctane, ethenyl-	10.678	1.272752
15	1,6-Octadiene, 2,5-dimethyl-, (E)-	11.135	0.28086
16	Citronellal	13.786	32.61177
17	2,6-Octadienal, 3,7-dimethyl-	13.968	0.896346
18	Citronellol	14.907	17.54064
19	Geraniol	15.384	25.94658
20	Hydrazine, 1-(5-hexenyl)-1-methyl-	15.566	0.830134
21	2-Undecanone	15.706	1.240976
22	2,6-Octadien-1-ol, 3,7-dimethyl-, formate, (E)-	15.893	0.211751
23	Octanenitrile	17.237	0.100424
24	1-Methylimidazole-4-carboxaldehyde	18.176	0.152756
25	Pentyl-Propyl ketone	18.731	0.097847
26	2,6-Octadien-1-ol, 3,7-dimethyl-, formate, (E)-	19.022	0.139448
27	1-10-di-epi-Cubenol	19.177	0.125103
28	Neric Acid	19.52	0.228993
29	Geranic acid	19.753	0.247908
30	Caryophyllene	20.215	0.301208
31	1,3-Cyclohexadiene-1-carboxaldehyde, 2,6,6-trimethyl-	20.469	0.130205
32	beta-Elemene	20.973	0.402101
33	Germacrene D	21.268	0.081891
34	Alpha-Caryophyllene	21.6	0.056572
35	Alpha-Humulene	21.725	0.05557
36	Naphthalene	22.617	0.08196
37	γ-Muurolene	22.861	0.246474
38	γ-Cadiene	23.235	0.099864
39	2-Tridecanone	23.806	1.818978
40	Cyclohexene, 1-methyl-4-(5-methyl-1-methylene-4-hexenyl)-, (S)-	24.07	0.133045
41	Naphthalene	24.195	0.363397
42	1-Formyl-2,2,6-trimethyl-3-cis-(3-methylbut-2-enyl)-5-cyclohexene	26.384	0.088152
43	Neral	26.509	0.07551
44	Caryophyllene oxide	27.017	0.462139
45	Alpha-Muurolol	28.19	0.217235
46	Cubenol	28.434	0.316996
47	Selina-6-en-4-ol	28.901	1.975845
48	1H-Cyclopropa[a]naphthalene	29.347	0.311006
49	.tau.-Cadinol	29.741	0.703909
50	2-Pentadecanone	32.356	0.160316

**Table 4:** Metabolites present in JCEO.

Metabolites	Aqueous Extract	Ethanolic Extract
Reducing sugar	+	+
Anthraquinones	+	+
Proteins and amino acids	−	+
Phlabotannins	−	+
Tannins	+	−
Resins	+	−
Terpenoids	+	+
Flavonoids	+	+
Quinones	+	+
Sterols and steroids	+	+
Diterpenes	−	+
Anthocyanins	−	−
Flavanones	+	+
Cardiac glycosides	− (Only brown ring)	+
Saponins	−	−
Phenols	+	+
Fixed oils and fatty acids	+	−

+: Presence, −: Absence.

**Table 5:** IC_50_ values of Java Citronella extracts.

Java Citronella Extract	IC_50_
Ethanolic extract	196.116 μg/mL
Aqueous extract	420 μg/mL

**Table 6:** Effect of incorporation of Java Citronella on visual mold growth on bread stored at room temperature, where DM = detected mold and NDM = no detected mold.

Samples	Day 0	Day 2	Day 4	Day 6	Day 8
Control	NDM	NDM	DM	DM	DM
C + JCP 0.5%	NDM	NDM	NDM	DM	DM
C + JCP 1%	NDM	NDM	NDM	DM	DM
C + JCP 1.5%	NDM	NDM	NDM	DM	DM
C + JCP 2%	NDM	NDM	NDM	NDM	DM
C + JCP 2.5%	NDM	NDM	NDM	NDM	DM
C + JCP 3%	NDM	NDM	NDM	NDM	DM

**Table 7:** Results obtained after the application of different % of JCEO on the weevils.

Concentration% of JCEO	Number of Insects in Dishes	30 min	6 h	24 h	48 h	60 h
2% C	10 LW	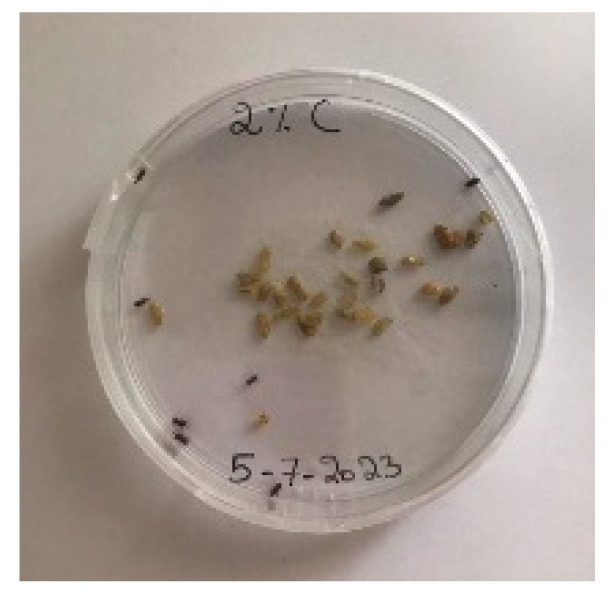 0 DW	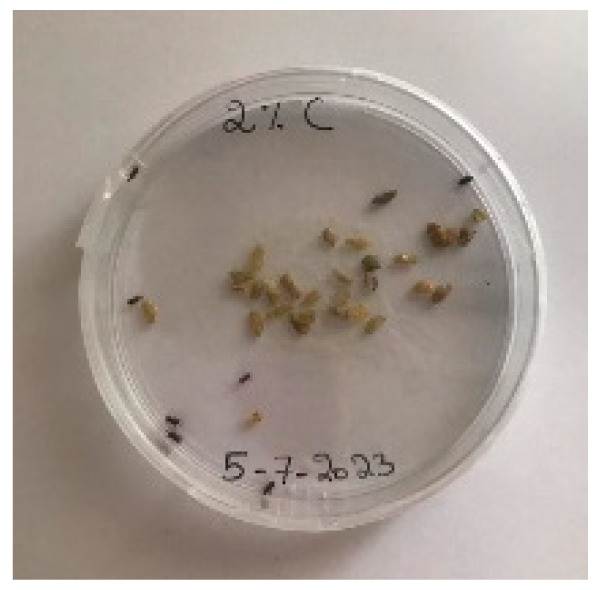 0 DW	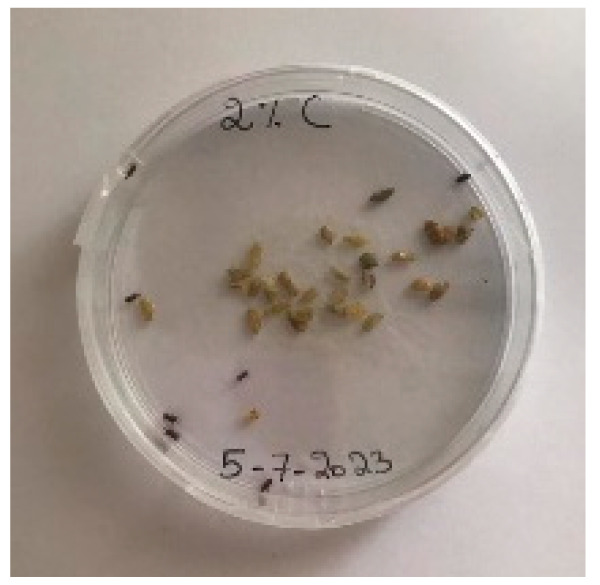 0 DW	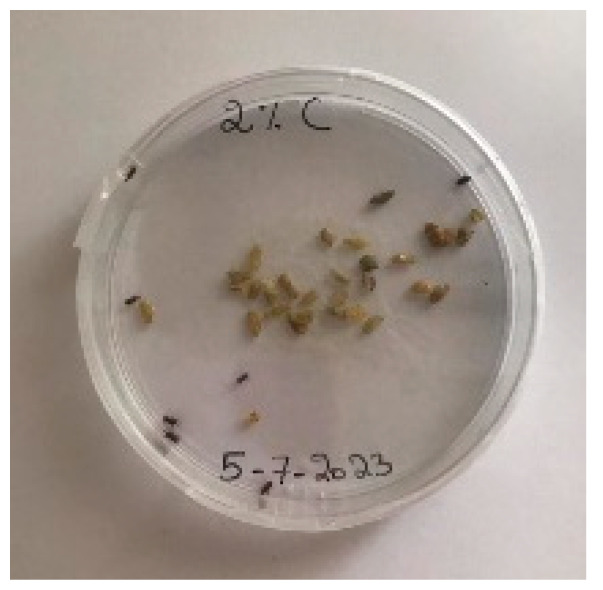 0 DW	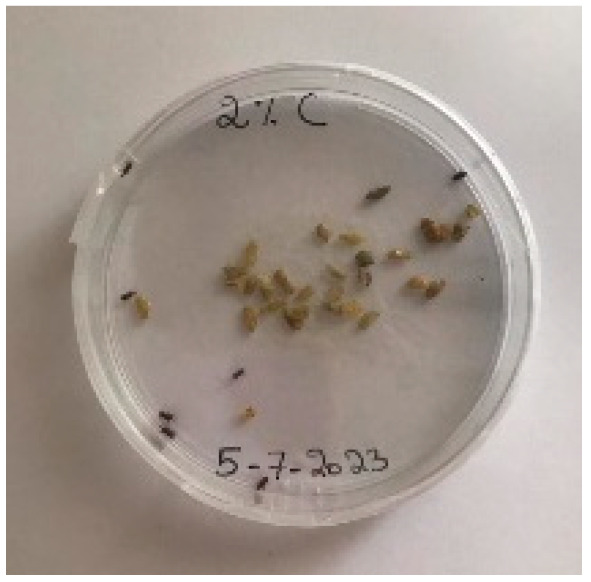 0 DW
3% C	10 LW	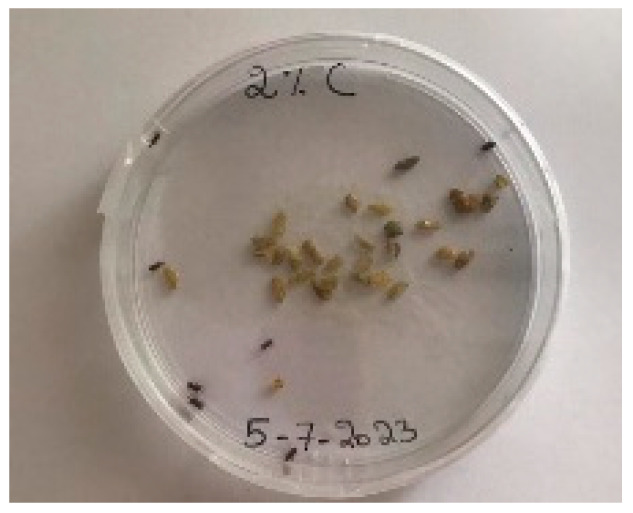 0 DW	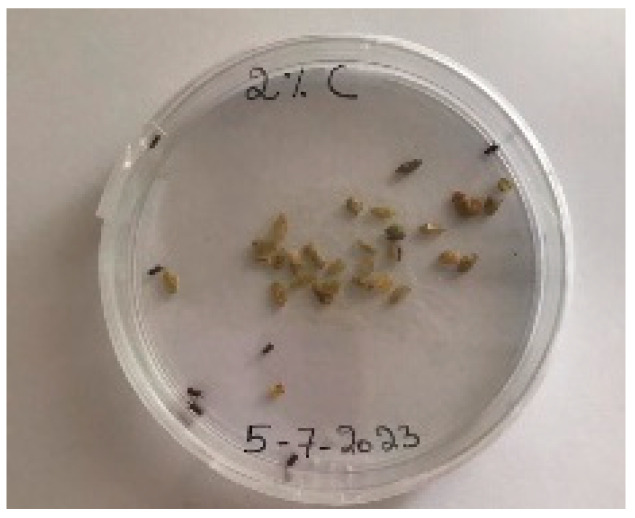 1 DW	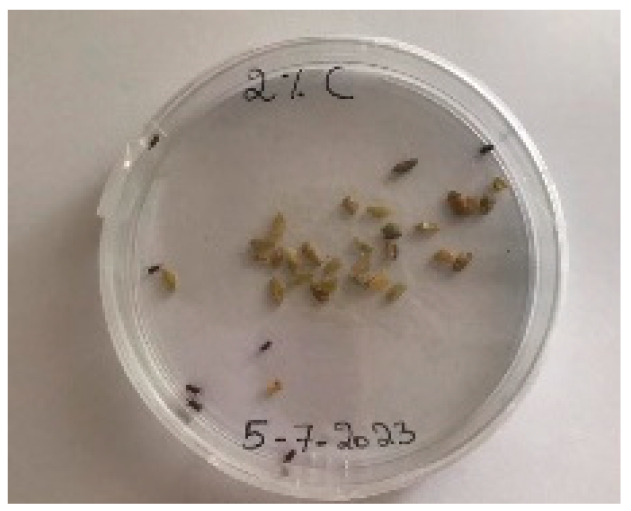 3 DW	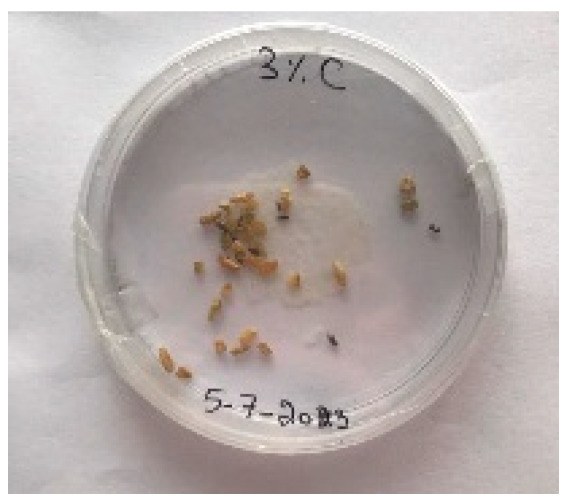 4 DW	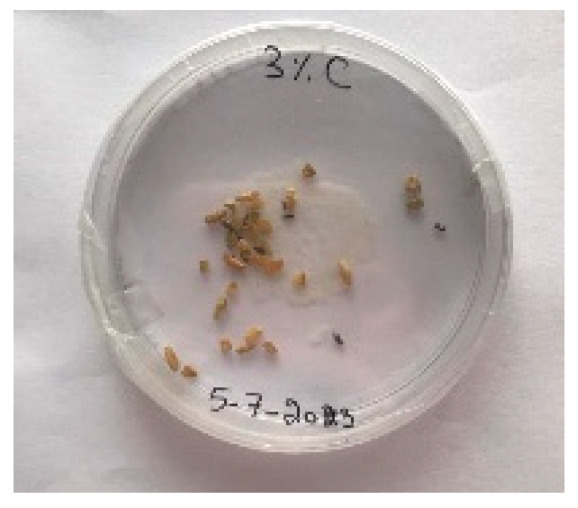 4 DW
5% C	10 LW	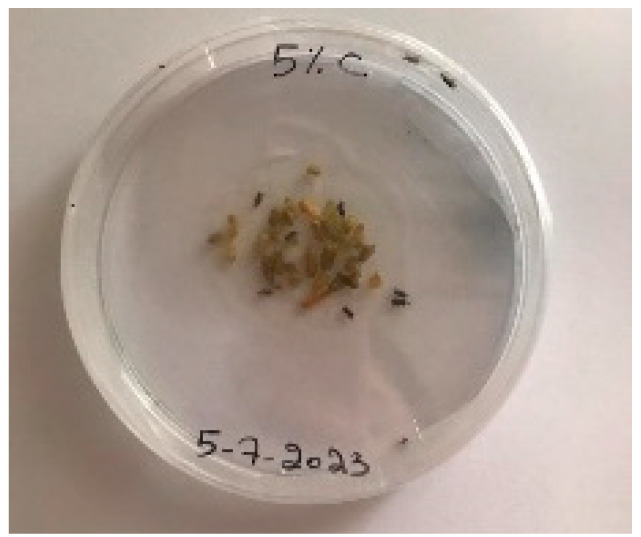 0 DW	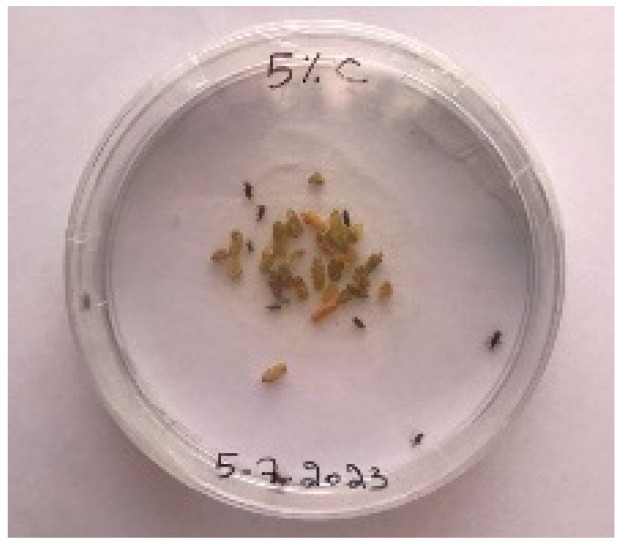 2 DW	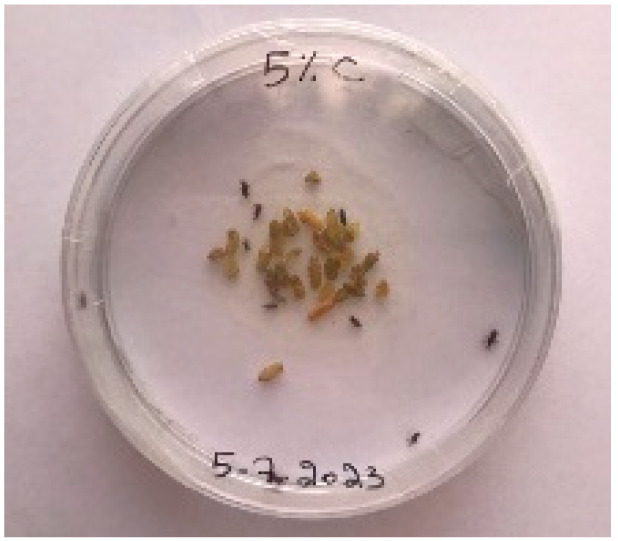 4 DW	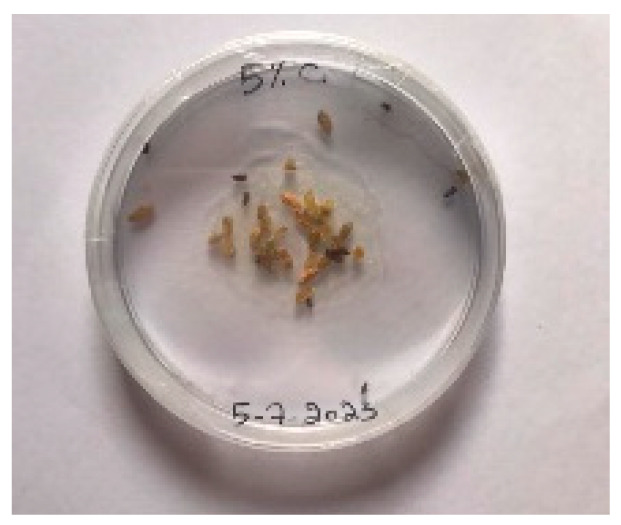 6 DW	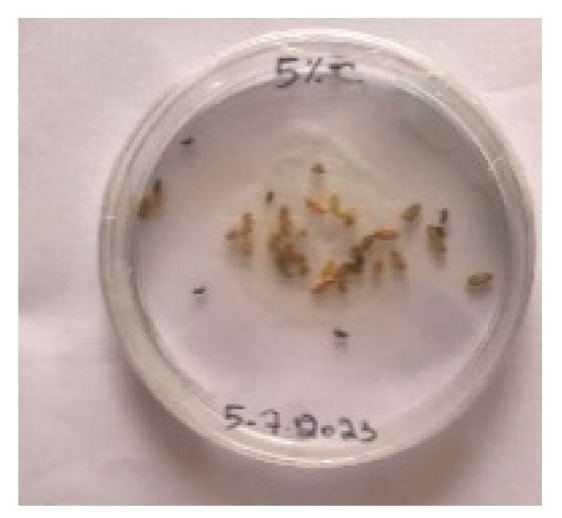 6 DW
7% C	10 LW	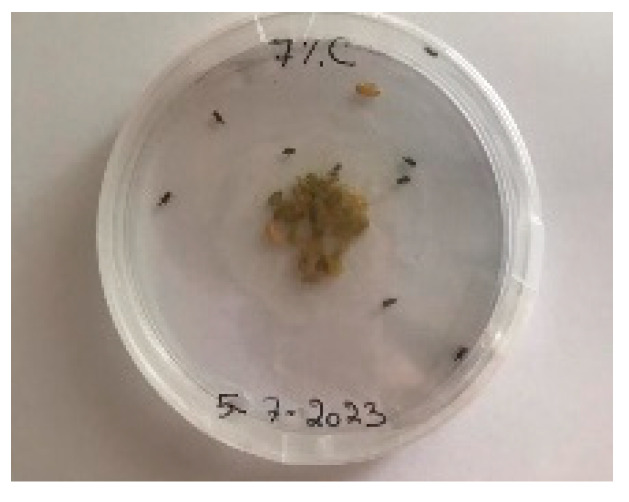 2 DW	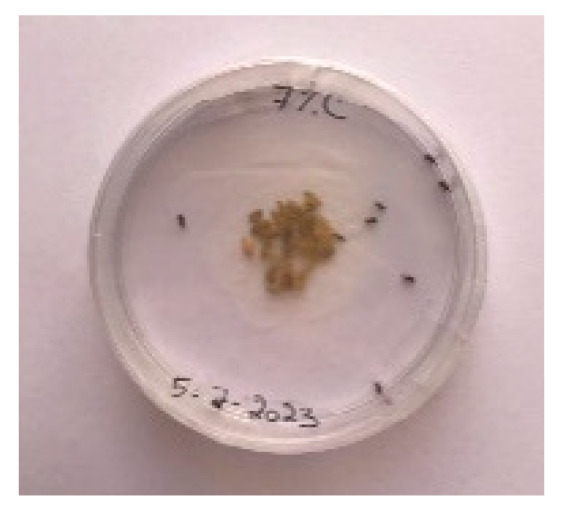 3 DW	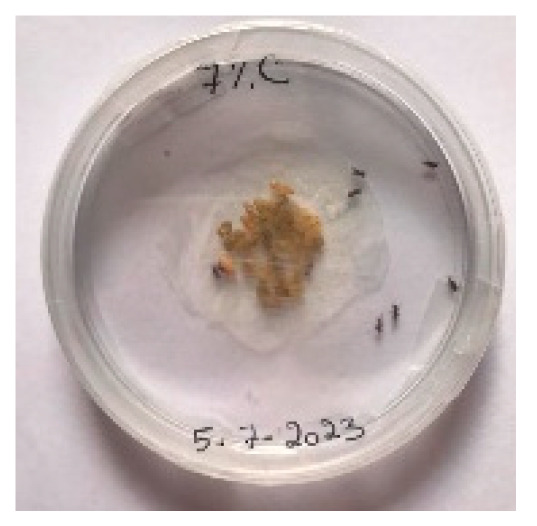 5 DW	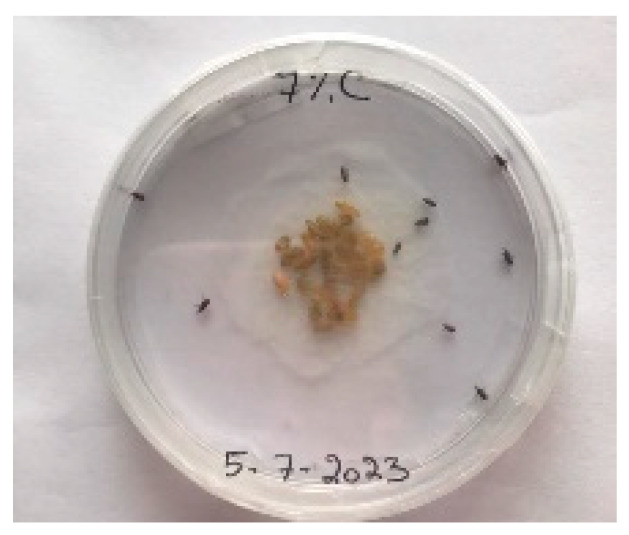 7 DW	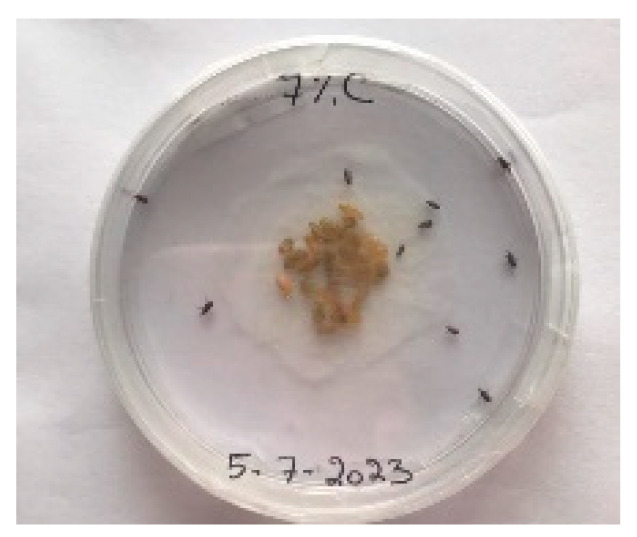 7 DW
10% C	10 LW	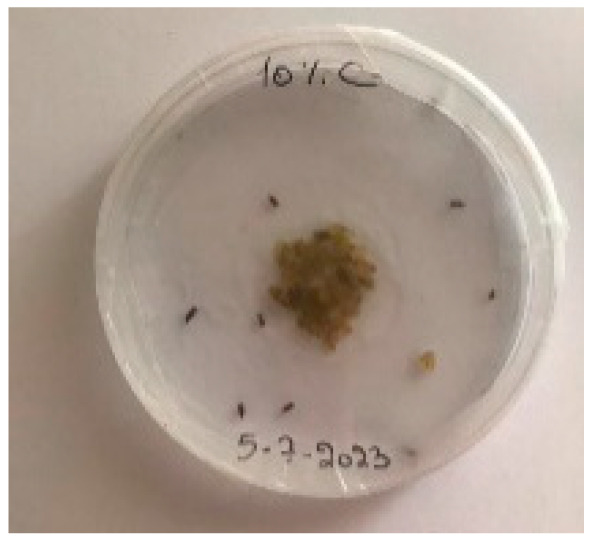 3 DW	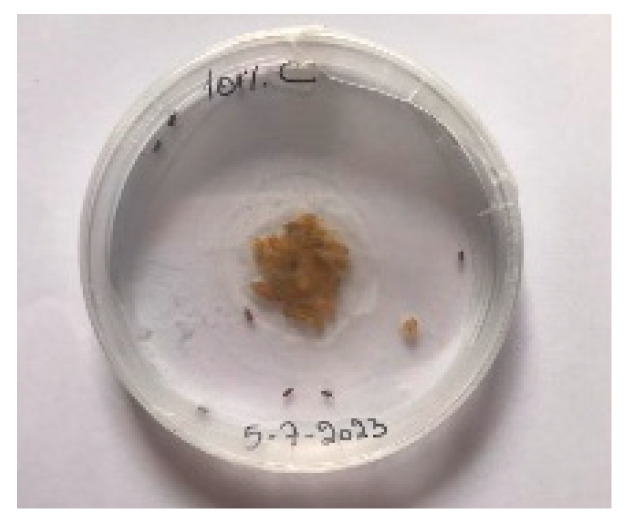 5 DW	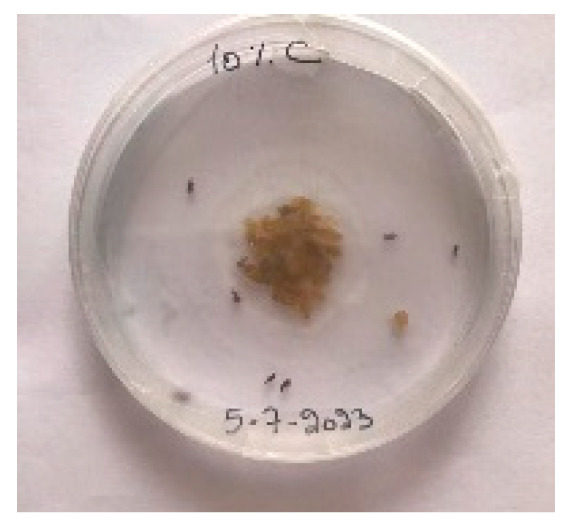 8 DW	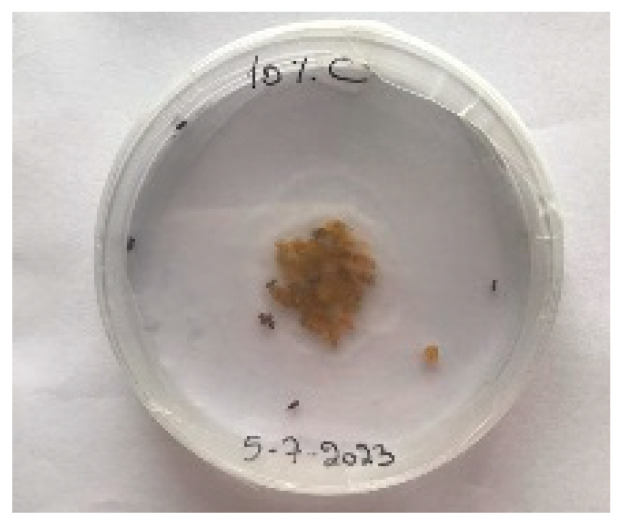 10 DW	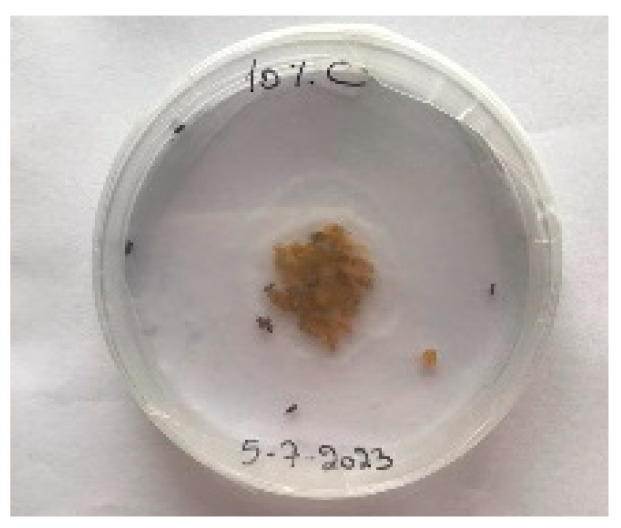 10 DW
NA	10 LW	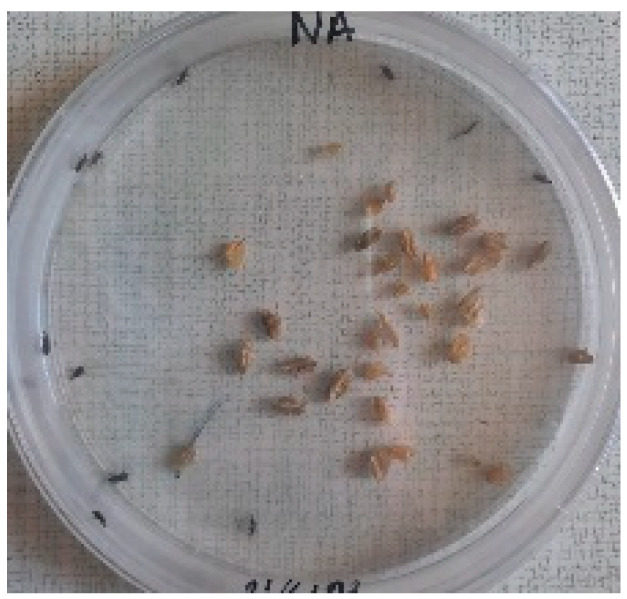 0 DW	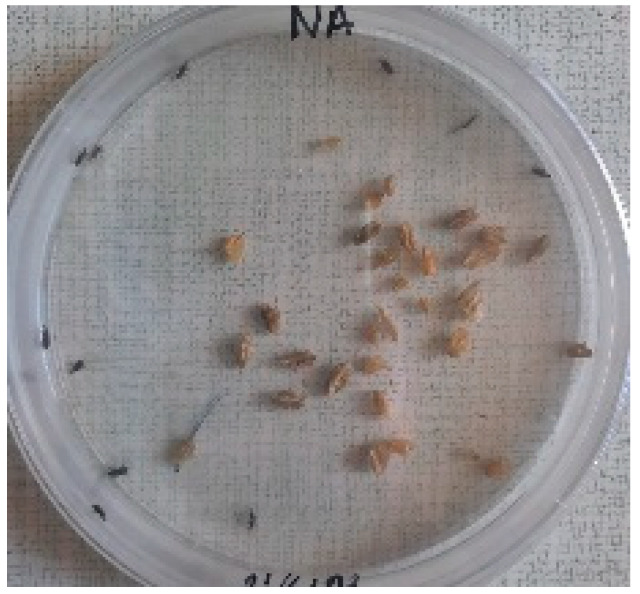 0 DW	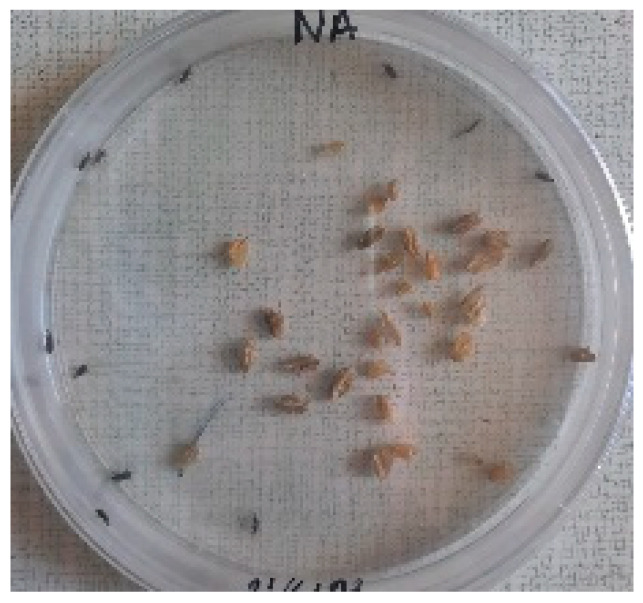 0 DW	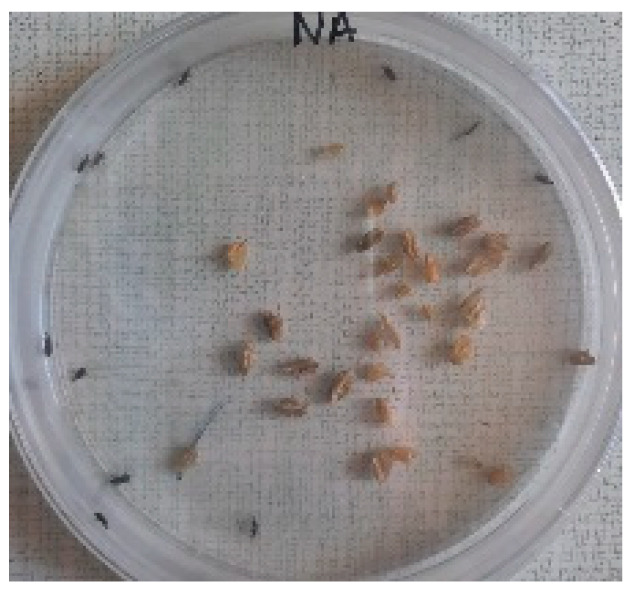 0 DW	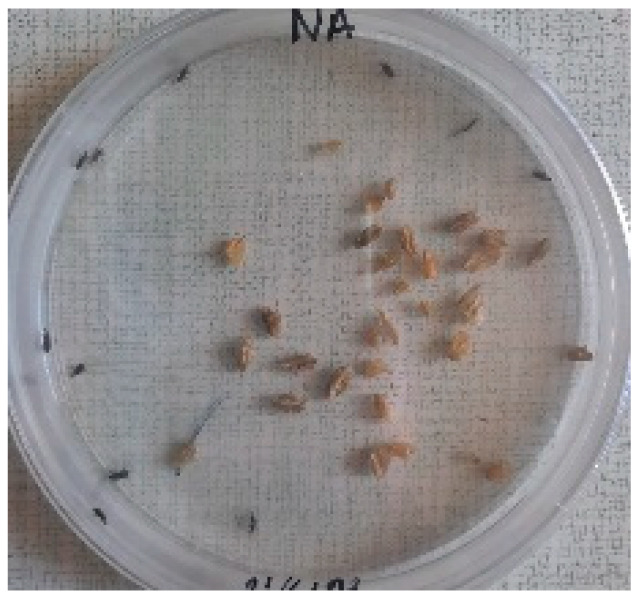 0 DW
Ethanol	10 LW	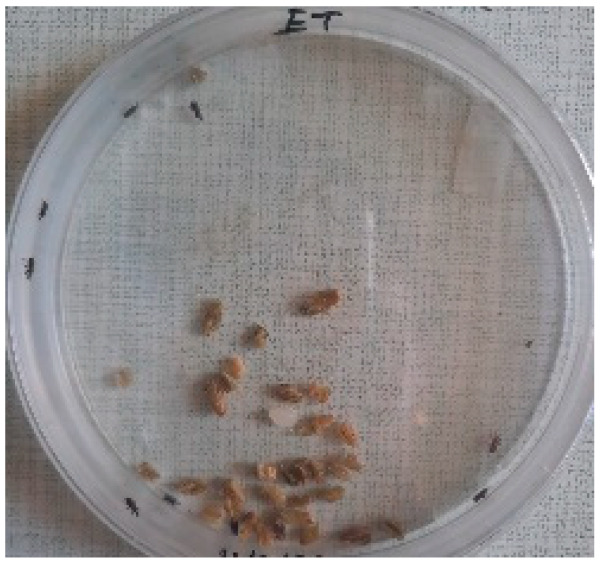 0 DW	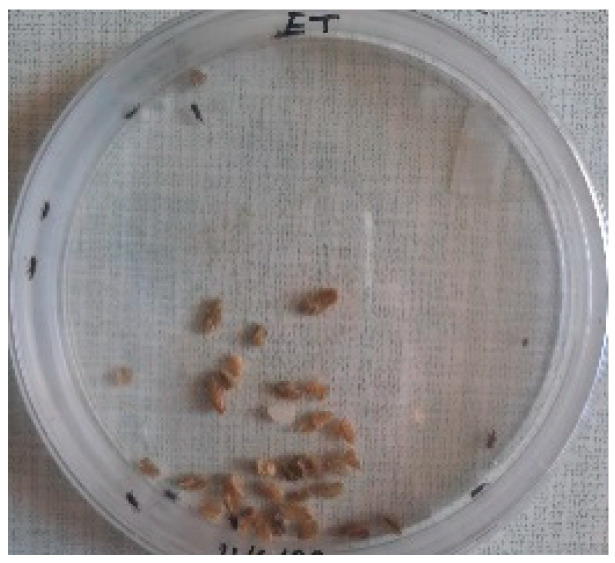 0 DW	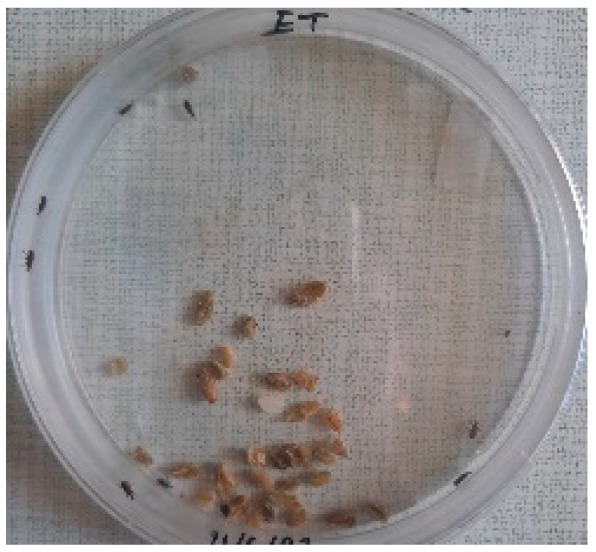 0 DW	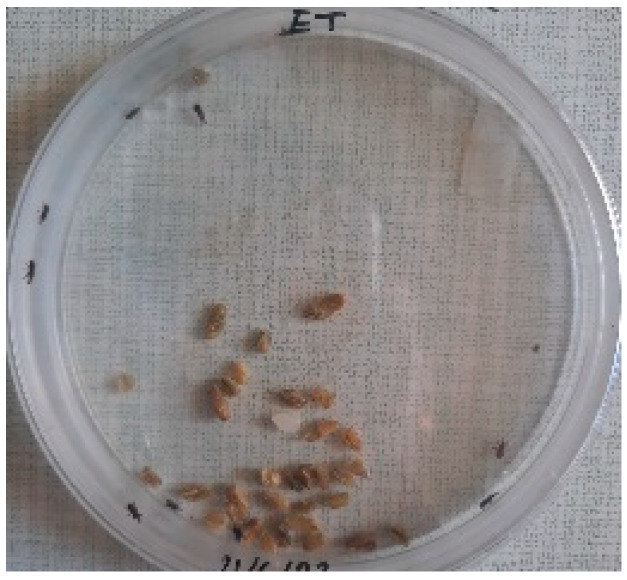 0 DW	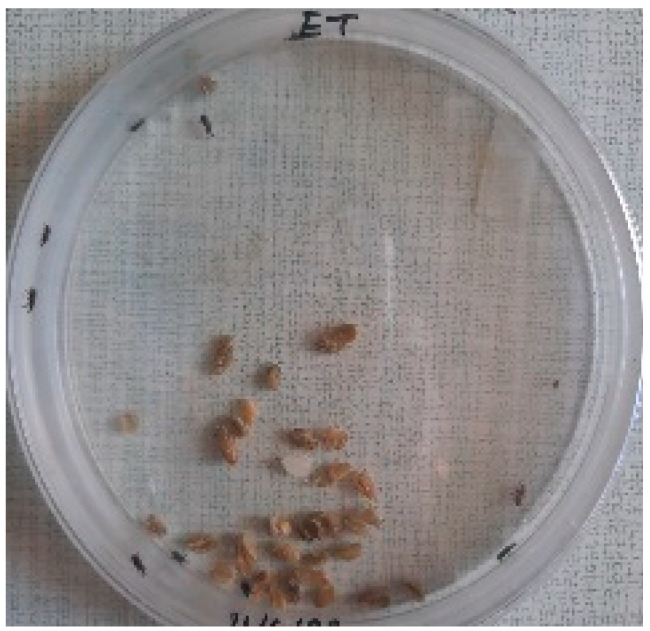 0 DW

**Table 8:** LT_50_ of JCEO at different percentages.

Dose of Extracts	LT_50_
2%	NA
3%	NA
5%	48 h
7%	24 h
10%	6 h

## Data Availability

The original contributions presented in the study are included in the article/Supplementary Materials, further inquiries can be directed to the corresponding authors.

## Supplementary Materials

The following supporting information can be downloaded at: https://www.mdpi.com/article/10.3390/foods13050803/s1, Table S1: Statistical analysis of the bread fortified with Java Citronella powder moisture content at day 1 by using oven drying method; Table S2: Statistical analysis of the bread fortified with Java Citronella powder moisture content at day 4 by using oven drying method; Table S3: Statistical analysis of the bread fortified with Java Citronella powder moisture content at day 8 by using oven drying method.
