# Supplementary material for: Cymbopogon winterianus (Java Citronella Plant): A Multi-Faceted Approach for Food Preservation, Insecticidal Effects, and Bread Application
Source: Foods. 2024 Mar 5;13(5):803. doi: 10.3390/foods13050803 (PMC10931247; doi:10.3390/foods13050803)
Supplement: Supplementary file 1 [file foods-13-00803-s001.zip › foods-2900439-supplementary.pdf]

Cymbopogon winterianus (Java Citronella Plant): A Multi-Faceted Approach for Food Preservation, Insecticidal Effects, and Bread Application

Marwa Rammal [1], Adnan Badran [2], Chaden Haidar [1], Abbas Sabbah [1], Mikhael Bechelany [3,4,*], Maya Awada [1], Khodor Haidar Hassan [1], Mohammad El-Dakdouki [5,*] and Mohamad T. Raad [6]

[1] Department of Food and Technology Studies, Faculty of Agronomy, Lebanese University, Beirut P.O. Box 146404, Lebanon; marwa.rammal.1@ul.edu.lb (M.R.); chaden.haidar@iul.edu.lb (C.H.); abbas.sabbah@gmail.com (A.S.); maya.awada19@gmail.com (M.A.); khodorhh@gmail.com (K.H.H.)
[2] Department of Nutrition, University of Petra Amman Jordan, Amman P.O. Box 961343, Jordan; abadran12@gmail.com
[3] Institut Européen des Membranes (IEM), UMR-5635, University of Montpellier, École Nationale Supérieure de Chimie de Montpellier (ENSCM), Centre National de la Recherche Scientifique (CNRS), Place Eugene Bataillon, 34095 Montpellier, France
[4] Functional Materials Group, Gulf University for Science and Technology (GUST), Mubarak Al-Abdullah 32093, Kuwait
[5] Department of Chemistry, Faculty of Science, Beirut Arab University, P.O. Box 11-5020, Riad El Solh, Beirut 11072809, Lebanon
[6] Department of Chemistry, Lebanese International University-Beirut (LIU), Salim Street, Mazraa, Beirut 146404, Lebanon; mohamad.raad01@liu.edu.lb
* Correspondence: mikhael.bechelany@umontpellier.fr (M.B.); m.eldakdouki@bau.edu.lb (M.E.-D.)

Post Hoc analysis: compare means, one way ANOVA

Table S1. Statistical analysis of the bread fortified with Java Citronella powder moisture content at day 1 by using oven dried method.

Effect	Repeated Measures Analysis of Variance (Spreadsheet4) Sigma-restricted parameterization Effective hypothesis decomposition				
	SS	Degr. of Freedom	MS	F	p
Intercept	26675.61	1	26675.61	66240.37	0.000015
Error	0.81	2	0.40		
Sample	1.10	6	0.18	1.70	0.203679
Error	1.29	12	0.11		

Table S2. Statistical analysis of the bread fortified with Java Citronella powder moisture content at day 4 by using oven dried method.

	SS	Degr. of Freedom	MS	F	p
Intercept	13519.17	1	13519.17	11787.07	0.000085
Error	2.29	2	1.15		
Sample	148.69	6	24.78	117.76	0.000000
Error	2.53	12	0.21		

Table S3. Statistical analysis of the bread fortified with Java Citronella powder moisture content at day 8 by using oven dried method.

	Repeated Measures Analysis of Variance (Spreadsheet4) Sigma-restricted parameterization Effective hypothesis decomposition				
Effect	SS	Degr. of Freedom	MS	F	p
Intercept	7059.177	1	7059.177	5378.996	0.000186
Error	2.625	2	1.312		
Sample	39.771	6	6.629	25.476	0.000004
Error	3.122	12	0.260		
